# Sensitivity to light in bipolar disorder: implications for research and clinical practice

**DOI:** 10.1192/bjp.2023.150

**Published:** 2024-05

**Authors:** Amber Roguski, Philipp Ritter, Daniel J. Smith

**Affiliations:** 1 Division of Psychiatry, University of Edinburgh, Edinburgh, UK; 2 Centre for Clinical Brain Sciences, University of Edinburgh, UK; 3 Clinic for Psychiatry and Psychotherapy, Carl Gustav Carus University Hospital, Technical University of Dresden, Dresden, Germany

## Abstract

Circadian dysfunction is a core feature of bipolar disorder and may be due, at least in part, to abnormalities of non-visual photoreception. We critically review the evidence for light hypersensitivity in bipolar disorder and discuss how this may shape future research and clinical innovation, with a focus on a possible novel mechanism of action for lithium.

For individuals with bipolar disorder - particularly those living at more extreme latitudes - episodes of mania and depression exhibit a sensitivity to seasonal patterns of light exposure. Analyses of hospital admission records demonstrate peaks for mania during spring/summer, coinciding with longer daylength (photoperiods) and peaks for depression in winter when daylight is more limited. Mania and depression admissions increase particularly around the spring and autumn equinoxes respectively, when day-to-day changes in photoperiod length are most rapid. Interestingly, these trends appear to be strongest for women, suggesting a possible sex difference in photoperiod sensitivity.

Seasonal variation in symptoms may provide a key mechanistic insight for circadian disruption in bipolar disorder: namely, that changes in light exposure levels can disrupt circadian rhythms and destabilise the euthymic mood state. According to this ‘light hypersensitivity hypothesis’, people with bipolar disorder may have some pathophysiological vulnerability that makes them more susceptible to the destabilising effects of excess light at night and rapid daily changes in photoperiod length. Indirect clinical evidence supporting light hypersensitivity in bipolar disorder comes from the recognised efficacy of chronotherapies such as bright-light therapy for bipolar depression and blue-light blocking glasses (or ‘dark therapies’) for mania (1).

Between the 1980s and early 2000s, several studies investigated light sensitivity in bipolar disorder by assessing *in vivo* light-induced melatonin suppression (for review see (2)). Guided by observations that melatonin levels may be elevated during mania and decreased during bipolar depressive episodes, initial investigations of light sensitivity assessed small numbers of people with bipolar disorder during extreme mood states (for example, two patients with mania and two with bipolar depression) (2) (Table 1). This small study reported 50% suppression of melatonin when bipolar disorder subjects were exposed to 500 lux (compared to minimal suppression in a control group). Increasing light intensity to 1500 lux almost completely suppressed melatonin levels in the bipolar disorder group, compared to only 60% suppression in the control group. These melatonin suppression experiments were subsequently extended to euthymic bipolar disorder, with several research groups reporting overall increased melatonin suppression in response to night-time light stimulation (2) (Table 1).

Conversely, several groups have reported that control subjects may have either greater or comparable levels of melatonin suppression to bipolar disorder (2). In one study by Nurnberger et al., this null result became positive when the bipolar disorder group was stratified into type I (BD-I) and type II (BD-II): individuals with BD-I exhibited greater melatonin suppression than BD-II and controls. Additionally, unmedicated bipolar disorder subjects had the greatest melatonin suppression. The largest and most well-controlled study to date by Ritter and colleagues used a well-defined BD-I cohort and found no evidence for light hypersensitivity or elevated melatonin suppression compared to controls (2).

These contradictory results raise several important issues. First, the within-group variation of melatonin suppression in these studies demonstrates that light sensitivity is not uniform. Indeed, we now know that sensitivity to light is influenced by age, sex, genetic variability, eye colour and pupil size, as well as by environmental factors such as prior light exposure. These factors mean that light hypersensitivity is enriched in certain demographic, at-risk and disease populations. For example, increased sensitivity to light (and elevated melatonin suppression) has been reported in younger age groups and in Delayed Sleep-Wake Phase Disorder, and some studies suggest that people with unipolar depression may have *reduced* sensitivity to light (2). This variability may also apply to subtypes of bipolar disorder: melatonin suppression may be greater in BD-I than in BD-II (but, as noted above, the only study to assess this so far had a very small sample size).

Another limitation of previous bipolar disorder light hypersensitivity investigations is the lack of in-depth phenotypic characterisation of study participants. For example, several studies were conducted prior to DSM-IV (1994), before BD-II was introduced and specific inclusion/exclusion criteria or diagnostic definitions were often not provided. It is also not clear whether the broad clinical spectrum of bipolar disorder (from BD-NOS to BD-II and BD-I) directly parallels a light sensitivity spectrum, or if other confounding factors (such as age, sex and medication) contribute to differences in light sensitivity between BD-I and BD-II. To date there has been no study which compares control participants with distinct BD-I and BD-II groups.

The evidence supporting light hypersensitivity in bipolar disorder therefore comes from a relatively limited number of small studies. Discrepancies between studies may be explained by confounding factors such as light delivery timing, differences in spectral composition, medication status, differences in pupillary diameter, bipolar disorder diagnostic heterogeneity and individual differences in light sensitivity. One important factor that no studies have adjusted for (including those by Ritter et al.) is individual differences in circadian phase. As people with bipolar disorder often exhibit delayed circadian phase, delivery of the light stimulus at the same time for all participants is likely to be a serious confound. Future experiments should therefore adjust the timing of light stimuli delivery according to an individual’s circadian phase.

It is also important to note that most investigations into light hypersensitivity were conducted prior to the discovery (in 1998) of melanopsin-containing intrinsically photosensitive retinal ganglion cells (ipRGC), which are the main conduit for non-visual/circadian light effects. These studies therefore lacked a key piece of mechanistic information, which might have informed study design decisions and the interpretation of findings.

Clearly more research on light sensitivity in bipolar disorder (and potential links to mechanisms of relapse) is warranted. Objective markers of light hypersensitivity to assess in future work would include: functional and structural changes in the retino-hypothalamic-pineal pathway; changes in melatonin levels; changes to behaviour; and changes in mood or activity symptoms in response to light stimuli. The success of light- and dark-chronotherapies - as well as experimental evidence that melatonin suppression recovery does not differ between bipolar disorder and control groups (2) - suggests that pathways that are downstream from the suprachiasmatic nucleus (SCN) for melatonin synthesis are intact and sufficient for entraining circadian rhythms, and that light is in itself capable of influencing mood states.

Some evidence therefore points to photic stimulation as a putative trigger for changes in circadian markers (including, but not limited to, melatonin secretion). A logical next step is to plan investigations at the level of the retina and, specifically, to study ipRGCs. There have been no cellular studies of ipRGC function in bipolar disorder so far. However, retinal imaging has identified that people with bipolar disorder may have decreased thickness of retinal nerve fibre and ganglion cell layers. Furthermore, psychophysical testing has found colour vision abnormalities in people with bipolar disorder. Retinal pathology may therefore be a key (but previously unrecognised) feature of bipolar disorder, and light hypersensitivity occurring via ipRGC dysfunction is feasible and testable.

Taking this a step further, some kind of pathological change to retinal structure and/or function could result in a vulnerability to changes in both visual and non-visual responses. One consequence of this is could be super-sensitivity to blue light at night and increased susceptibility to circadian phase shifts (as demonstrated by (3)) caused by natural or synthetic light stimuli that are out-of-sync with the external environment. These phase shifts could lead to misalignment of internal and external clocks and disrupted daily rhythms of sleep, neuroplasticity, activity, metabolism and immune function. The adverse effects of these changes (such as sleep fragmentation and sleep deprivation) could then cause individuals to relapse into mania or depression. Importantly for this tentative hypothesis, circadian dysfunction in bipolar disorder is posited as a downstream consequence of retinal pathology.

If light hypersensitivity is a true biomarker of bipolar disorder (even within a sub-group) then the implications for understanding current treatments and for developing new therapies could be substantial. Lithium is a gold standard treatment for bipolar disorder but is not effective for at least one third of patients, suggesting the existence of sub-groups of ‘lithium-responsive’ and ‘lithium non-responsive’ bipolar disorder. Although lithium’s mechanism of action is unclear, good evidence indicates that it regulates neurotransmission and has neuroprotective effects by reducing neural excitotoxicity. It is also known that lithium has a dose-dependent effect on circadian period length and can stabilise internal free-running period length, as well as delaying sleep phase. We would add that lithium may also be effective (at least in some patients) by correcting retinal hypersensitivity to light at night.

The plausibility of retinal effects of lithium is supported by human MRI research which shows that lithium accumulates within the eyes (4). Early animal studies demonstrated that lithium stabilised circadian rhythms by reducing light-induced phase delays and that it influenced pupillary response to light (but not dark). Thus, it is likely that lithium acts by modulating the retinal response to light rather than modulating pupillary muscle tone. In healthy human participants, five days of lithium therapy reversed the effects of light-induced melatonin suppression (2). One study using electrooculography and electroretinography found no retinal electrophysiological differences in response to light between bipolar disorder participants on long-term lithium therapy and controls (5). Although the authors concluded that light sensitivity was not a feature of bipolar disorder and that lithium did not reduce light hypersensitivity, in fact the opposite may be true. It is theoretically possible that the long-term lithium treatment of the bipolar participants (mean 7 years) may have restored light sensitivity levels to that of the control participants.

Overall, these studies tentatively support a mechanism of action for lithium that reduces light hypersensitivity. There have been no attempts to date to classify people with bipolar disorder according to light sensitivity levels. Additionally, research now needs to establish how light sensitivity changes across different bipolar mood states. To do so could be a first step towards providing treatments which specifically target light hypersensitivity sub-groups, with flexibility to account for dynamic, mood-dependent light sensitivity profiles.

If people with bipolar disorder can indeed be stratified according to light sensitivity, research investigating the mechanisms of action of different therapies within these sub-groups could move forward. Longer-term, this stratification approach could inform personalised medicine (and specific chronotherapeutic) approaches to treating bipolar disorder.

As a clinical and research community, we need to communicate more clearly to patients and their families that chronotherapies are useful and feasible and we need to work with patients to produce high quality, accessible bipolar-specific information on light, circadian disruption, sleep and rest/activity rhythms.

## Conclusion

Converging evidence from basic science and from clinical practice suggests that light hypersensitivity may be a biomarker of risk for relapse, at least in a sub-group of patients with bipolar disorder. A light hypersensitivity perspective of bipolar disorder is an exciting focus for future research. It confers challenges for both patients and clinicians but also new opportunities for patient stratification and treatment innovation.

## Figures and Tables

**Table 1:** Summary of melatonin suppression studies in bipolar disorder. *Ritter et al. 2020 used photon density (1.6*1013 photons/cm^2^/s) as a measure of photic stimulation.

Evidence for bipolar disorder light hypersensitivity					
Authors	Bipolar disorder group (n; state)	Control group (n)	Bipolar disorder medications	Bipolar disorder group melatonin suppression (%)	Control group melatonin suppression (%)
Lewy et al. 1981	n=4; 2 manic, 2 depressed	n=6	No details	500 lux: 50%	500 lux: negligible
				1500 lux: ~100%	1500 lux: 60%
Lewy et al. 1985	n=11; euthymic	n=24	Medications ceased 2 weeks prior to study. Most participants were on lithium	500 lux: 61.5 ± 1.6%	500 lux: 28% ± 5.8%
Nathan et al. 1999	n=8; presumed euthymic	n=63	6/8 on lithium medication. 2 not on medication	200 lux: 43.57%	200 lux: 14.33%
Hallam et al. 2009	n=7 (BD-I); euthymic	n=33	Stable medications, no further details	200 lux: 51%	200 lux: ~25%
				500 lux: 61%	500 lux: ~30%
				1000 lux: 73%	1000 lux: ~45%
**Inconclusive evidence for bipolar disorder light hypersensitivity**					
**Authors**	**Bipolar disorder group (n; state)**	**Control group (n)**	**Bipolar disorder medications**	**Bipolar disorder group melatonin suppression (%)**	**Control group melatonin suppression (%)**
Nurnberger et al. 2000	n=29 (n=21 BD-I, n=8 BD-II); euthymic	n=50	Mix of medications	500 lux: 29.8% ± 5.5% *BD-I only 62.7%±15.6%	500 lux: 34.6% ± 2.6% *matched to BD-I only 40.0%±9.1%
**Evidence against bipolar disorder light hypersensitivity**					
**Authors**	**Bipolar disorder group (n; state)**	**Control group (n)**	**Bipolar disorder medications**	**Bipolar disorder group melatonin suppression (%)**	**Control group melatonin suppression (%)**
Lam et al. 1990	n=8 (n=4 BDI-I, n=4 BD-II); 2 BD-I depressed, 2 BD-I manic, 4 BD-II depressed	n=15	No psychotropic drugs	No % given; control had larger suppression	No % given; control had larger suppression
Whalley et al. 1991	n=15; euthymic	n=15	No medications	500 lux: 38.3% ± 8.2SE	500 lux: 50.4% ± 6.5SE
Ritter et al. 2020*	n=33 (BD-I); euthymic	n=57	Mixed medications	5.0% (SD: 39.1%)	14.6% (SD: 20.6%)
